# Estimation of three-dimensional chromatin morphology for nuclear classification and characterisation

**DOI:** 10.1038/s41598-021-82985-9

**Published:** 2021-02-09

**Authors:** Priyanka Rana, Arcot Sowmya, Erik Meijering, Yang Song

**Affiliations:** 1 School of Computer Science and Engineering, University of New South Wales, Sydney NSW, Australia; 2 Graduate School of Biomedical Engineering, University of New South Wales, Sydney NSW, Australia

**Keywords:** Cellular imaging, Image processing, Machine learning

## Abstract

Classification and characterisation of cellular morphological states are vital for understanding cell differentiation, development, proliferation and diverse pathological conditions. As the onset of morphological changes transpires following genetic alterations in the chromatin configuration inside the nucleus, the nuclear texture as one of the low-level properties if detected and quantified accurately has the potential to provide insights on nuclear organisation and enable early diagnosis and prognosis. This study presents a three dimensional (3D) nuclear texture description method for cell nucleus classification and variation measurement in chromatin patterns on the transition to another phenotypic state. The proposed approach includes third plane information using hyperplanes into the design of the Sorted Random Projections (SRP) texture feature and is evaluated on publicly available 3D image datasets of human fibroblast and human prostate cancer cell lines obtained from the Statistics Online Computational Resource. Results show that 3D SRP and 3D Local Binary Pattern provide better classification results than other feature descriptors. In addition, the proposed metrics based on 3D SRP validate the change in intensity and aggregation of heterochromatin on transition to another state and characterise the intermediate and ultimate phenotypic states.

## Introduction

Intercellular interactions within a tissue microenvironment take place through diverse mechanical and biochemical signals that control cellular development, differentiation and homeostasis^1–4^. Various studies have revealed that a derangement in signals disrupts chromatin dynamics and commences differential regulation of gene expression and genomic imbalance that subsequently triggers oncogenic transitions^2,5,6^. Chromatin domains on mutation undergo disarrangement of heterochromatin (HC) (condensed chromatin) organisation and observe coarsening or opening of HC, resulting in an increase or loss of HC aggregates all around the nuclear matrix region, respectively^6–8^. This transformation is closely associated with metastatic potential and holds diagnostic and prognostic significance. At the molecular level, alterations appear as a change in nuclear texture formed by wrinkles, folds and trenches manifested through entwined strands of nuclear proteins, lamins and chromatin. Changes in nuclear texture occur in conjunction with other morphological variations such as nuclear and nucleolar size, shape and count at the tissue level and the organisation of protein content, based on which cancerous cells are differentiated from normal ones^2,7^. Although these alterations have been a gold standard for late-stage diagnosis of tumours, the description of their origin, interdependency and progression is not clear; therefore early diagnosis of cancer, drug discovery and prognosis care remains a challenge^2,7,9^.

The nucleus, being a prominent organelle of eukaryotic cells, houses cellular DNA (chromatin), hosts chromosome formation and offers a dynamic research domain to measure and study nuclear morphological changes from normal to malignant cells correlated with genetic alterations. In cross disciplines such as mechanobiology, nuclear morphological quantification has emerged as a promising approach to study the effect of external signals on nuclear morphology and their further impact on enclosed protein organisation. Quantitative analysis of variations in nuclear morphology and protein configuration helps to explain the mechanisms underlying cellular alterations and has opened new avenues for curative models in cancer care^2,10,11^.

Quantitative models are usually evaluated on classification problems to comprehend and measure nuclear morphological alterations. Machine learning techniques that extract handcrafted features are often chosen over deep learning as the latter does not provide interpretable features^2^. Research on novel handcrafted feature descriptors thus remains active despite the success of deep learning^12–15^, as they perform competently for well-defined problems and do not require massive amounts of data for training. Cellular changes at the molecular level can be understood through an adequate feature description capable of capturing low-level details of cells in multi-dimensions. Nuclear texture, being one of the low-level feature when described and quantified accurately in 3D, has the potential to provide insights that enable early diagnosis and prognosis. Recent developments in confocal microscopy have enabled more effective in-vivo intraoperative studies through 3D fluorescence images, to analyse the heterogeneity of cellular patterns^16^.

**Figure 1:**
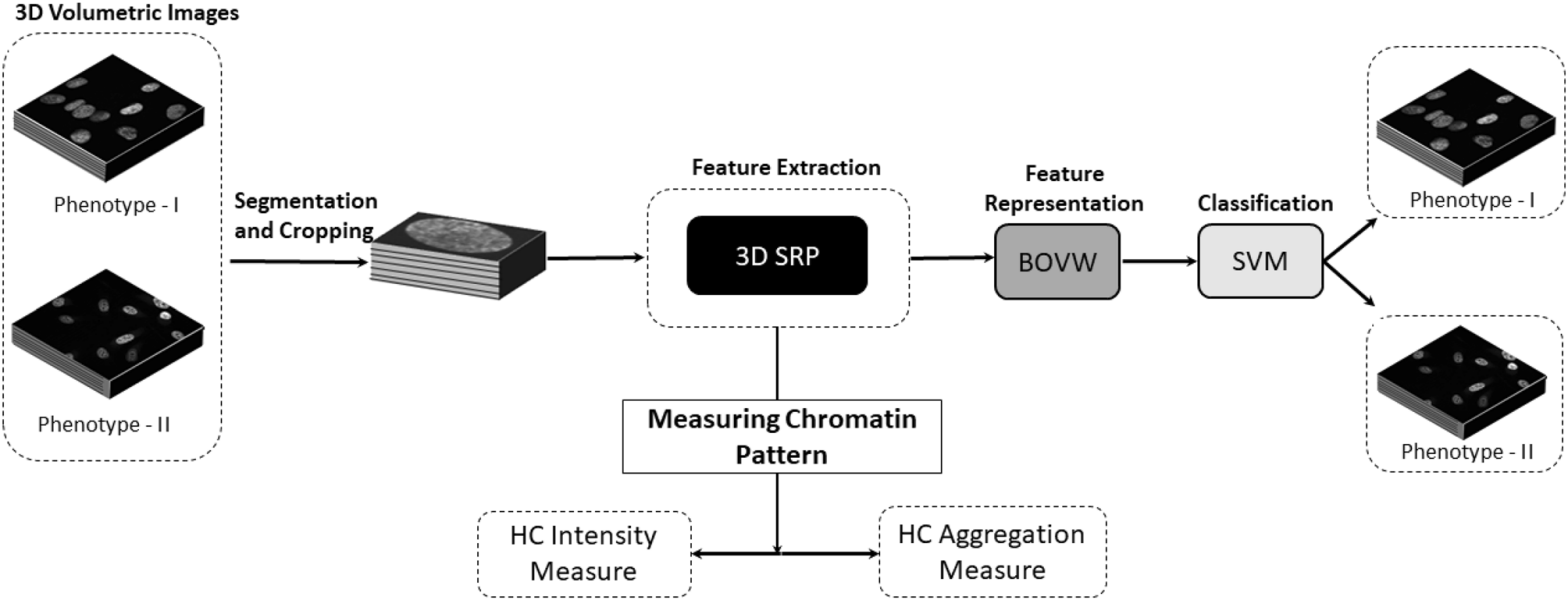
Pipeline to classify cellular phenotypic states and measure chromatin patterns. Cells are segmented and cropped, followed by extraction of cellular texture features using proposed 3D SRP. Image-based classification is performed using the linear model trained on extracted features. Following classification, heterochromatin intensity and aggregation is measured using the proposed 3D SRP. The presented model is evaluated on publicly available 3D image datasets of human fibroblast and human prostate cancer cells.

Various studies have established the discriminative performance of 3D texture analysis more than 2D^17–19^. In the early years of 3D texture description, Gray-Level Co-Occurrence Matrix (GLCM) descriptors were widely used for fluorescence microscopy images^19^. Local Binary Pattern-Three Orthogonal Planes (LBP-TOP)^20^ has been considered as the leading approach for 3D biomedical images^21^. It has outperformed commonly used, diverse variants of 3D texture descriptors such as Moments, Haralick, Tamura, Gabor and other variants of LBP for cell classification^13,21,22^. LBP-TOP computes standard LBP features from three orthogonal planes separately and concatenate them to represent 3D LBP feature descriptors. Apart from LBP-TOP, the recently proposed 3D RSurf^13^ uses spherical coordinates with diverse combinations of azimuthal and polar angle to analyse 3D images. The latest scale and rotational invariant 3D Scale-Invariant Feature Transform (SIFT)^23^ extracts texture features from a spherical image window, and is employed to target high intensity points of DAPI (4’,6-diamidino-2-phenylindole) images that represent HC.

In this study, we propose a novel approach to compute third plane information for texture description using cubic patches and hyperplanes within the patch. The proposed approach is adapted to the existing SRP feature descriptor^14^ which describes the distribution of intensities in diverse patterns, originally defined for texture description of 2D images. SRP is based on the dimensionality-reduction technique known as Random Projections (RP), which compresses high dimensional data, captures salient information without information loss and preserves inter-distance of data values while projecting to a lower dimensional space. It is a rotationally invariant texture feature descriptor and has achieved competitive results and efficiency. The proposed 3D approach extends SRP by extracting features from the third plane using hyperplanes built in the cubic patches of the volumetric image (explained in “Methods”). Bag of Visual Words (BOVW)^15^ is then applied to generate the final 3D feature descriptor.

The proposed 3D SRP is used to measure changes in the chromatin patterns and perform cell nucleus classification, and evaluated on two publicly available 3D image datasets of DAPI stained nuclei of human fibroblast and human prostate cancer cells^10,11^ (Fig. 1). Images are preprocessed following quality protocols and segmented to obtain individual cells with minimal background. Cellular features are extracted and further represented using BOVW to reduce redundant features and obtain a global feature vector to represent each class features. Subsequently, a linear Support Vector Machine (SVM) model is trained to classify the phenotypes. Following classification, 3D SRP features are computed to quantify changes in HC on transition from one phenotype to another. The changes in the intensity and aggregation of HC illustrate epigenetic mechanisms.

Along with the earlier mentioned handcrafted features (SIFT, LBP and RSurf), Convolutional Neural Networks (CNNs)^24^ are also used to generate deep learning features for comparison. The significance of including third plane information for low-resolution volumetric images is also investigated by comparing the performance of 3D texture descriptor with its respective pseudo 3D form that ignores the interslice intensity correlations. The results demonstrate 3D SRP is one of the most effective texture feature description methods for this data set, and has potential to measure variations in chromatin patterns.

## Results and discussions

### 3D SRP texture description classifies phenotypic states with best results

Proposed 3D SRP is evaluated for the classification of 3D images of DAPI stained nuclei of two different cell collections, each comprised of different states with distinct morphological features for binary classification. The first cell collection has 3D volumetric images of primary human fibroblast cells in two phenotypic states/classes: (1) proliferating fibroblasts (*PROLIF*), and (2) cell cycle synchronised by the serum-starvation protocol (*SS*) (see Supplementary Table S1 and Fig. S1). The second cell line has 3D volumetric images of human prostate cancer cells (PC3) in two states/classes: (1) epithelial (*EPI*) state, and (2) mesenchymal transition (*EMT*) state (see Supplementary Table S1 and Fig. S1). PC3 phenotypic states exhibit different and quantifiable nuclear morphological features that are useful in studying prostate cancer progression.

Cellular alterations at the molecular level may happen inconsistently in a small group of defective cells in a tissue microenvironment. Therefore, image classification based on cellular feature descriptors is recommended to comprehend the variations. Performance of 3D SRP is compared with deep learning features (Fisher vector based CNN features (FV-CNN), details in the “Methods” section) and other handcrafted feature descriptors, which employ distinct approaches for third plane inclusion and are comparatively recent, such as RSurf, and the widely utilised LBP and SIFT texture descriptors. The choice of LBP and SIFT is also motivated from a recent review of texture descriptors that mentions SIFT and LBP as milestone texture feature descriptors^15^.

**Figure 2:**
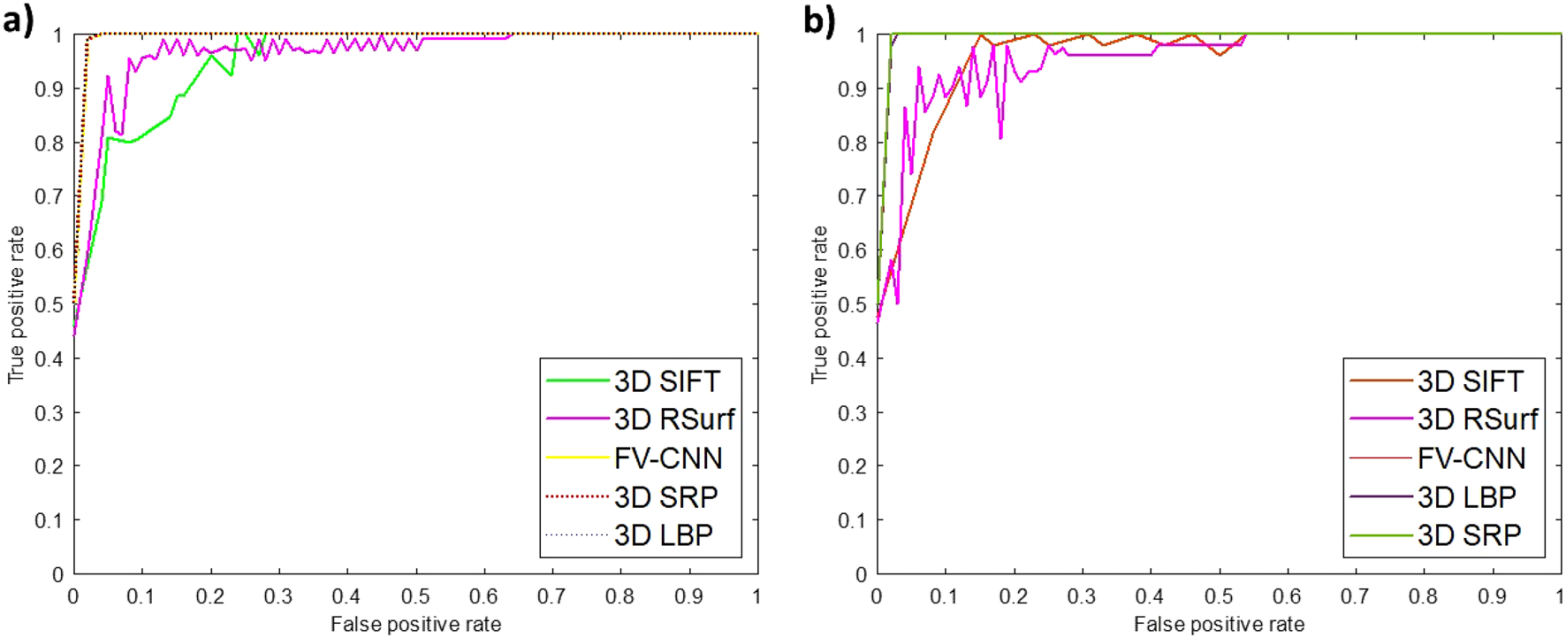
ROC curves for all feature descriptors. (**a**) Fibroblast dataset. (**b**) PC3 dataset. Note that 3D SRP, 3D LBP and FV-CNN curves are overlapping in (**a**). 3D LBP and FV-CNN curves are overlapping in (**b**).

The results shown in Table 1 are obtained via 10-fold cross-validation (explained in “Methods”). Classification performance is evaluated using AUC (Area-Under-the-Curve) of the ROC (Receiver-Operating-Characteristic) curve and the F1 score. Figure 2 demonstrates performance of classification models for all the folds through the ROC plots. Classification models are also compared statistically for their metric values. As shown in Table 1, the mean AUC and F1 score from the 10-fold cross-validation scheme have different standard deviation values for feature descriptors. Therefore, the Kruskal–Wallis test^25^ is employed, which is a non-parametric method also called one-way ANOVA based on ranks, to compare the means of more than two sample groups. The hypothesis for the Kruskal–Wallis test is:

1 $$\begin{aligned} \textit{Null hypothesis }{H_0}= \textit{All classification models have the same mean rank.} \end{aligned}$$

**Table 1:** Classification results for Fibroblasts and PC3 Cell Collection.

Features	Fibroblast cell line				PC3 cell line			
	AUC	F1 score	Precision	Recall	AUC	F1 score	Precision	Recall
3D SRP	${\textbf{0.997}} \pm {\textbf{0.003}}$	${\textbf{0.99}} \pm {\textbf{0.02}}$	${\textbf{0.98}} \pm {\textbf{0.03}}$	${\textbf{0.998}} \pm {\textbf{0.002}}$	${\textbf{0.999}} \pm {\textbf{0.002}}$	${\textbf{0.98}} \pm {\textbf{0.02}}$	${\textbf{0.97}} \pm {\textbf{0.003}}$	${\textbf{0.98}} \pm {\textbf{0.03}}$
Pseudo 3D SRP	$0.94 \pm 0.04$	$0.94 \pm 0.04$	$0.97 \pm 0.04$	$0.91 \pm 0.06$	$0.98 \pm 0.01$	$0.85 \pm 0.20$	$0.75 \pm 0.32$	$0.94 \pm 0.08$
3D LBP	${\textbf{0.997}} \pm {\textbf{0.003}}$	${\textbf{0.99}} \pm {\textbf{0.02}}$	${\textbf{0.98}} \pm {\textbf{0.03}}$	${\textbf{0.996}} \pm {\textbf{0.001}}$	$0.994 \pm 0.004$	$0.97 \pm 0.03$	$0.97 \pm 0.04$	$0.97 \pm 0.04$
Pseudo 3D LBP	$0.88 \pm 0.13$	$0.79 \pm 0.15$	$0.77 \pm 0.18$	$0.82 \pm 0.10$	$0.97 \pm 0.02$	$0.90 \pm 0.06$	$0.84 \pm 0.11$	$0.97 \pm 0.04$
3D SIFT	$0.96 \pm 0.02$	$0.90 \pm 0.05$	$0.90 \pm 0.06$	$0.91 \pm 0.13$	$0.98 \pm 0.03$	$0.96 \pm 0.06$	$0.97 \pm 0.04$	$0.94 \pm 0.07$
Pseudo 3D SIFT	$0.85 \pm 0.04$	$0.74 \pm 0.08$	$0.69 \pm 0.10$	$0.79 \pm 0.08$	$0.93 \pm 0.02$	$0.80 \pm 0.13$	$0.84 \pm 0.21$	$0.80 \pm 0.05$
3D RSurf	$0.98 \pm 0.02$	$0.92 \pm 0.03$	$0.93 \pm 0.04$	$0.92 \pm 0.02$	$0.97 \pm 0.03$	$0.88 \pm 0.09$	$0.85 \pm 0.12$	$0.93 \pm 0.09$
Pseudo 3D RSurf	$0.94 \pm 0.02$	$0.86 \pm 0.02$	$0.87 \pm 0.05$	$0.85 \pm 0.05$	$0.97 \pm 0.03$	$0.89 \pm 0.05$	$0.89 \pm 0.01$	$0.91 \pm 0.08$
FV-CNN	$0.996 \pm 0.004$	$0.93 \pm 0.16$	$0.90 \pm 0.25$	$0.989 \pm 0.01$	$0.995 \pm 0.01$	$0.96 \pm 0.06$	$0.95 \pm 0.03$	$0.96 \pm 0.08$
C-FC	$0.72 \pm 0.08$	$0.62 \pm 0.08$	$0.61 \pm 0.11$	$0.65 \pm 0.08$	$0.76 \pm 0.05$	$0.72 \pm 0.04$	$0.70 \pm 0.06$	$0.76 \pm 0.08$

Metric values of pseudo 3D feature descriptors and features obtained from the penultimate fully-connected layer (C-FC) of the CNN model are also included for comparison.

**Figure 3:**
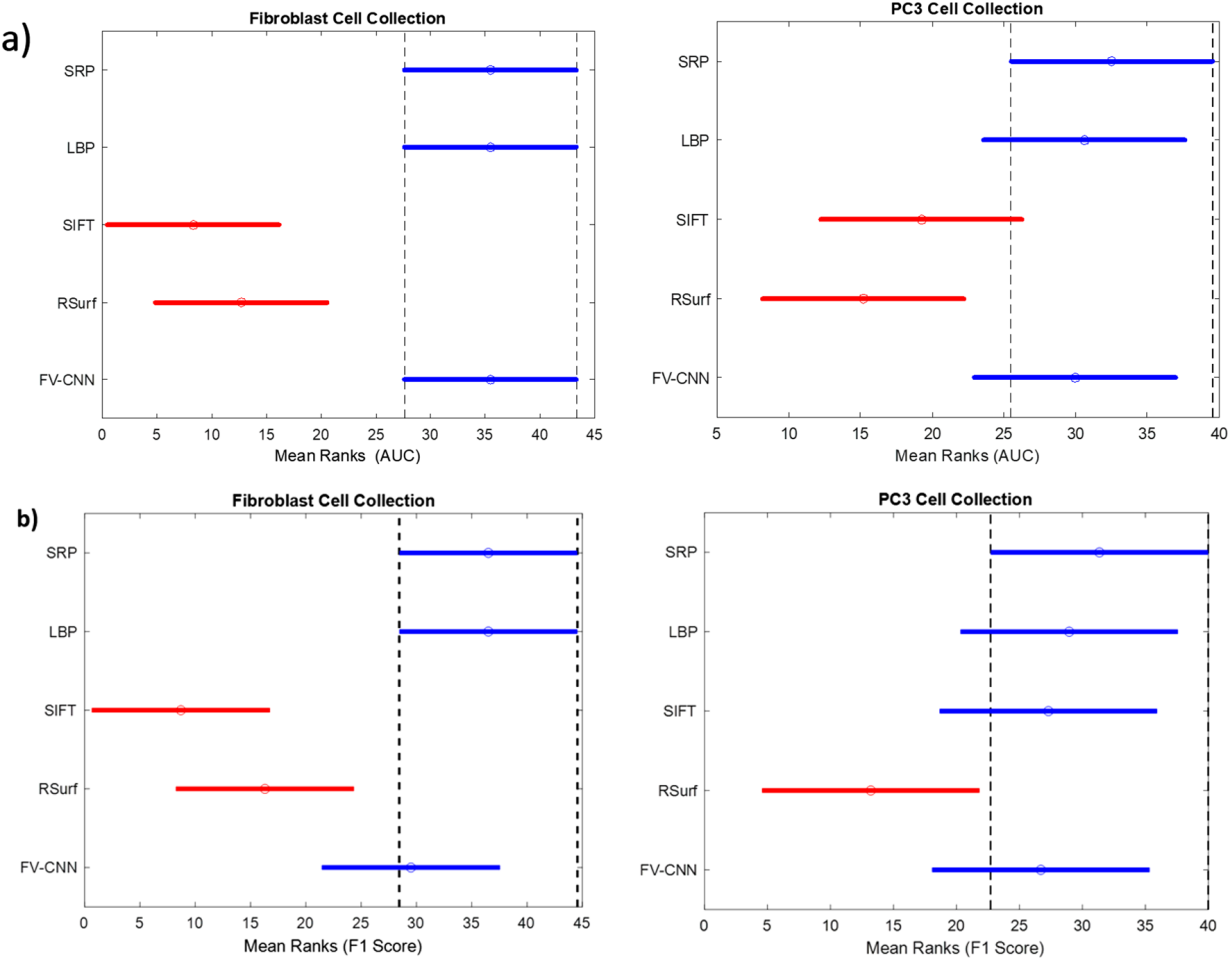
Mean ranks of 3D handcrafted descriptors and FV-CNN from Kruskal–Wallis test results for fibroblast and PC3 cell collections (**a**) AUC. (**b**) F1 Score.

At 1% significance level and very small p-value (< 0.01), the Kruskal-Wallis test indicates the difference between mean ranks of five groups (SRP, LBP, SIFT, RSurf, FV-CNN). In Fig. 3, different colours of the lines indicate different mean ranks and population. The length of the line represents the comparison interval, and the extent of overlap of lines implies the range of similarity of corresponding groups.

As demonstrated in Fig. 3a,b, 3D LBP, 3D SRP and FV-CNN are at similar ranks and from a separate population than 3D RSurf and 3D SIFT. Although the mean F1 score for FV-CNN is lower than 3D SRP and 3D LBP for fibroblast cell images, statistical results demonstrate their performance to be similar (Fig. 3b). This is because the F1 score of two subsamples deteriorated the mean FV-CNN. However, all other subsamples performed as well as 3D SRP and 3D LBP. F1 scores for most of the feature descriptors are good for the PC3 data set with a high number of slices (Fig. 3b). Overall performance evaluation implies that 3D SRP and 3D LBP performed better than all other considered handcrafted feature descriptors, while 3D SRP achieved better results than 3D LBP for the PC3 data set. In addition, SRP features offer more robust feature description as these are obtained by encoding intensity distributions of an image patch in square, circular and inter ring structure, while LBP utilises only pixel differences in circular pattern. In comparison, FV-CNN features show an overlapping ROC curve with 3D SRP and 3D LBP and demonstrate distinctly higher performance than C-FC.

### 3D SRP measures changes in chromatin pattern and characterises phenotypic states

Chromatin patterns vary according to cell cycle phase, developmental state and chromatin positioning^26^. The changes in the chromatin density are owing to DNA methylation and histone acetylation, which are major epigenetic mechanisms and highly relevant to human development and diseases^27^. Both processes share a reciprocal relationship, where DNA methylation is enriched in transcriptionally inactive heterochromatin, while histone acetylation is associated with transcriptionally active (or potentially active) euchromatin^27^. Epigenetic factors influencing gene expressions at the molecular level dictate cellular morphology and behaviours. When downregulation of gene expressions occurs, some of the euchromatin changes to heterochromatin that makes the segments of DNA more compact (DNA methylation)^28^. This is a reversible process; hence when active transcription is required, heterochromatin is converted to euchromatin which is lightly packed (histone acetylation)^28^. Thus, these conversions lead to the remodelling of chromatin structure inside the nucleus.

HC is characterised by the constriction in centromere, defined by a variant of histone H3, centromere protein-A (CENP-A) which is an established biomarker^29^. Being a profoundly condensed and compact chromatin fraction, it is easily detectable by DAPI staining^30,31^. Corresponding to the high luminance contrast regions in DAPI images, in previous studies HC is identified by applying a threshold equal to the sum of the minimum intensity and sixty per cent of the difference between the maximum and minimum intensities^9^. Following the proposed 3D SRP, this study takes two approaches based on pixel values (Eq. 11) and adjacent pixel differences (Eq. 12), to determine the chromatin pattern alterations in 3D. Heterochromatin to euchromatin ratio (*HC*/*EC*) of both cell states is computed as explained in Methods and statistically compared to inspect the nature of changes in chromatin pattern on transition to another phenotypic state. The two-sided Wilcoxon rank-sum test at the significance level of 1% is utilised to measure the difference in $HC/EC_{PixelValues}$ and $HC/EC_{PixelDifferences}$ between two classes for both cell lines. The corresponding null hypothesis is:

2 $$\begin{aligned} {H_{0\_Fibroblast}}= & {} \textit{Normal cell state has higher HC/EC than another phenotypic state} \end{aligned}$$

3 $$\begin{aligned} {H_{0\_PC3}}= & {} \textit{Normal cell state has lower HC/EC than another phenotypic state.} \end{aligned}$$

**Table 2:** Results from the two-sided Wilcoxon rank-sum test evaluating HC/EC and indicating the change in chromatin patterns on transition from normal (*PROLIF*, *EPI*) to another phenotypic states.

Cell groups	${{H}_{0}}$	${{HC/EC}_{PixelValues}}$	${{HC/EC}_{PixelDifference}}$	Conclusion
PROLIF-SS	(2)	$1.1{\text{ e }}{-}07$	$3.4{\text{ e }}{-}08$	Rejects $H_0$
EPI-EMT	(3)	$7.3{\text{ e }}{-}11$	$1.1{\text{ e }}{-}20$	Rejects $H_0$
EPI-EMT$^{179}$	(3)	$2.1{\text{ e }}{-}23$	$8.2{\text{ e }}{-}43$	Rejects $H_0$
EPI-EMT*	(3)	0.50	0.26	Accepts $H_0$

As shown in Table 2, the results from the test verify the statistical significance of the difference in $HC/EC_{PixelValues}$ and $HC/EC_{PixelDifferences}$ between two classes and indicate both ratios are higher for *SS* cells than for *PROLIF* (normal state) cells, while they are lower for *EMT* cells than for *EPI* (normal state) cells. Notably, low p-values obtained for statistical analysis of *HC*/*EC* ratios indicate the significant increase in intensity and HC aggregation, signifying HC condensation on the transition to *SS* state (see Supplementary Figs. S2 and S6). For the PC3 data set, on the other hand, there is a decrease in the intensity and HC aggregation, which signifies a decondensation or “open chromatin” state on the transition to the *EMT* state (see Supplementary Figs. S2 and S6). Changes in the chromatin pattern as implied by results in Table 2 are in accordance with other relevant studies^2,7,9,17^.

Note that the utilised data set has multiple subsets of images for each class where each subset represents one ‘run’ of the microscope of different cell samples. The test was conducted for all individual subsets, and the observations remained the same for all subsets except in one instance when the test was performed excluding one image subset (coded as 179 ($EMT^{179}$)) comprising 25 volumetric images with 134 cells in the *EMT* class. $EMT*$ refers to the image set without $EMT^{179}$. It consists of 117 cells and on evaluation, we observed high p-values at the significance level of 1% which indicates insufficient evidence to reject the null hypothesis for both ratios. However, the *z*-value (value of the *z*-statistic) of 0.6341 indicates a negative shift in the median of $HC/EC_{PixelDifferences}$ from *EPI* to *EMT* at 1% significance level.

Since *EMT* is a reversible transition from epithelial to mesenchymal state in which cells do not necessarily exist in ‘pure’ epithelial or mesenchymal state and comprise of multiple intermediate cell states (*ICS*) with hybrid features of pure states^32^. Therefore, based on obtained results (Table 2), it is suspected that available EMT images consists of cells in two cellular states. While many cells in $EMT*$ subset are in the *ICS*, cells in $EMT^{179}$ are in pure mesenchymal state or in *ICS* close to pure mesenchymal state. Corresponding p-values and z-values characterise cells in $EMT*$ as being of similar intensity but with slightly decondensed HC than in *EPI* state and cells in $EMT^{179}$ as being of lower intensity and highly decondensed HC than in *EPI* and $EMT*$ cells (see Supplementary Figs. S2 and S6).

Previously, the existence of two such *EMT* *ICS* has been found in mammary epithelium cells^33^. *EMT* cells with their *ICS* are of high importance in studies related to comprehension of cancer progression and drug resistance. Recently Sha et al.^32^ highlighted the characterisation and classification of *ICS* as a significant challenge and opportunity in the *EMT* field, as they are believed to play a vital role in disease progression and should be considered crucial biological entities. At present single-cell surface marker/RNA sequencing (scRNA-Seq) is the accepted method to identify the intermediate states that occur during *EMT* in metastasis. According to the obtained results, the proposed metrics based on SRP can be utilised to identify and characterise the *ICS* in *EMT* state of human prostate cancer cells. Even though images in *EMT* class consist of cells with diverse characteristics, good classification results are still achieved because of feature representation techniques (BOVW and FV), which derive new image features corresponding to the most dominant original features of the whole data set.

The *HC*/*EC* ratio was also computed following the same method with the original pixel values without random projections, and it was observed that the obtained p-value was higher. However, the inference remains the same for the pixel differences approach and no statistical significance in ratio difference is found for the pixel value method. This is because RP achieves compact representation of high dimensional data and preserves its inter-distances when projecting values to a lower dimensional space. This intensifies the salient information of the data, providing global features, and therefore, statistical analysis demonstrates the distinction of ratios more significantly. *HC*/*EC* ratios were also computed following the pseudo 3D approach. Similar to the proposed 3D method, the obtained results demonstrate a positive shift in *HC*/*EC* ratios from *PROLIF* to *SS* class and negative shift from *EPI* to *EMT* class. However, resultant p-values are higher than the values obtained from the proposed 3D approach, suggesting the latter is more credible.

**Figure 4:**
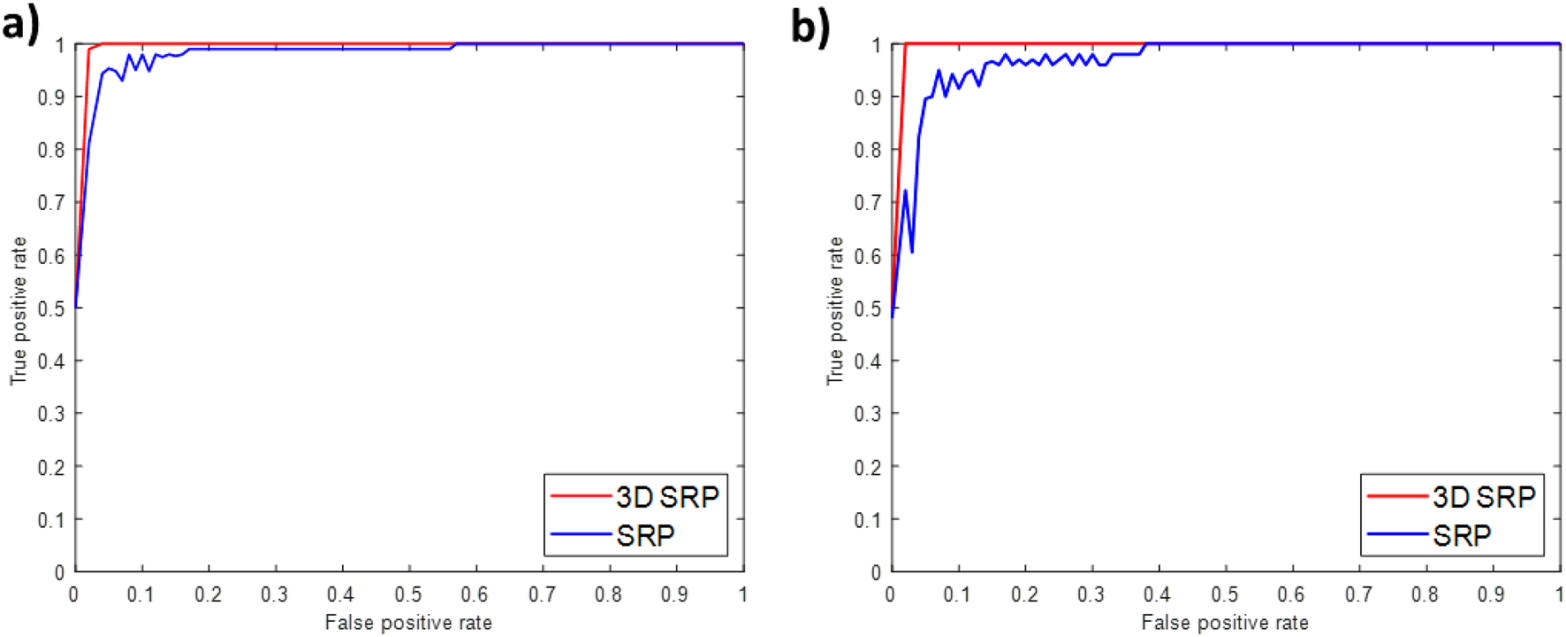
ROC curves: 3D SRP vs Pseudo 3D SRP (**a**) Fibroblast dataset. (**b**) PC3 dataset.

**Table 3:** Two sample t-test results comparing 3D and pseudo 3D texture descriptors.

Features	Fibroblast cell line				PC3 cell line			
	AUC		F1 score		AUC		F1 score	
	p-value	Conclusion	p-value	Conclusion	p-value	Conclusion	p-value	Conclusion
SRP	0.01	Rejects $H_0$	0.002	Rejects $H_0$	0.02	Rejects $H_0$	0.02	Rejects $H_0$
LBP	0.007	Rejects $H_0$	0.002	Rejects $H_0$	0.004	Rejects $H_0$	0.004	Rejects $H_0$
SIFT	0.0004	Rejects $H_0$	0.001	Rejects $H_0$	0.02	Rejects $H_0$	0.005	Rejects $H_0$
RSurf	0.008	Rejects $H_0$	0.004	Rejects $H_0$	0.48	Accepts $H_0$	0.69	Accepts $H_0$

### 3D texture description performs better with third plane inclusion

To study the impact of computing third plane information for low resolution, 3D versions of SIFT, LBP, RSurf and SRP was compared with their corresponding pseudo forms that ignores the interslice intensity correlations. The two-sample t-test at 5% significance level was used to compare AUC and F1 scores of pseudo and non-pseudo descriptors based on the null hypothesis:

4 $$\begin{aligned} \textit{Null hypothesis }{H_0}= \textit{Pseudo 3D outperforms 3D.} \end{aligned}$$

In addition, variability in performance differences between pseudo and non-pseudo versions of the feature descriptor was evaluated for all the folds through the ROC plots provided in Fig. 4 and Supplementary Figs. S4 and S5.

Although the 3D image data has low resolution along the *Z* plane, results demonstrate the advantage of utilising 3D feature descriptors for classification over their corresponding pseudo versions (Table 1). As shown in Table 3, at 5% significance level the test results verify the statistical significance of the advantage of non-pseudo versions of descriptors over the pseudo versions successfully except pseudo RSurf which is as good as non-pseudo 3D RSurf for the PC3 data set.

On comparing the performance of a feature descriptor on two data sets (fibroblast and PC3 cell collections), it is observed that the approach to include third plane information and the corresponding input parameters are significantly correlated with the number of slices in the volumetric image and must be carefully considered to obtain the best classification performance. For example, 3D SIFT based on spherical image window exhibited better performance for PC3 data set images with a higher number of slices, as the slices were adequate to build an appropriate sphere. In contrast, 3D RSurf’s performance deteriorated, as the considered number of polar angles were not sufficient to extract all the pixel information. Similarly, 3D SRP outperformed 3D LBP on the PC3 data set, as its capability to extract extensive information through hyperplanes was fully utilised for the images with a higher number of slices.

### Comparison with the state-of-the-art studies and correlation of nuclear texture and shape-based features

In the current study, obtained metric values based on texture description are higher than the state-of-the-art (SOTA) works obtained by classification using shape-based features on the same dataset. In Table 4, the best achieved results are shown in comparison to the SOTA^10,11^. High performance in the current study is credited to factors such as quality protocols in the preprocessing step, the segmentation approach and feature representation. Features are extracted from the segmented cell objects instead of whole image to avoid dilution of texture description by the image background and noise. Application of feature encoding techniques such as BOVW and Fisher Vectors reduced redundant features.

While the current work evaluates texture descriptors using 10-fold cross-validation, the compared SOTA works performed 20 split Leave-2-Opposite-Groups-Out (L2OGO) cross-validation. Due to the different approach adopted in this work for segmentation and quality protocols, the number of cell objects obtained is different from SOTA (see Supplementary Table S2). The SOTA works also merged features from all the channels by quantifying nucleoli-level features for each nucleus using statistical values such as average, median, maximum and higher moments, while the current study is focussed only on the DAPI channel, as the number of pixels from other channels is too small to be considered for texture description

**Table 4:** Comparison of classification results with existing studies.

Reference	Cross-validation scheme	Fibroblast cell line		PC3 cell line	
		AUC	F1 score	AUC	F1 score
Kalinin et al.^10^	20 split-L2OGO	$0.81 \pm 0.25$	$0.71 \pm 0.19$	$0.77 \pm 0.017$	$0.82 \pm 0.018$
Kalinin et al.^11^	L2OGO	$0.899 \pm 0.123$	Precision : $0.92 \pm 0.115$ Sensitivity: $0.87 \pm 0.224$	$0.76 \pm 0.224$	Precision : $0.81 \pm 0.334$ Sensitivity: $0.62 \pm 0.447$
Current study (3D SRP)	10-fold	$0.997 \pm 0.003$	$0.99 \pm 0.02$	$0.999 \pm 0.002$	$0.98 \pm 0.02$

As evident from earlier studies^10,11^ on the same dataset, G0/G1 Serum Starvation Protocol imposes significant changes in cell size and shape, which refer to changes in lamins, the protein primarily responsible for nuclear size and shape. The presented experiments demonstrate changes in intrinsic texture mainly formed by chromatin. Previous studies have demonstrated the variations in chromatin pattern with nuclear size changes^34^. Accordingly, our work also indicates the molecular linkage of lamins and chromatin proteins and the correlation of the textural changes with shape and size alterations.

## Methods

### Data description

In this study 3D Cell Nuclear Morphology Microscopy Imaging Dataset is used; the largest available public 3D image set obtained from Statistics Online Computational Resource (SOCR)^10,11^. All cell images have three channels showing different fluorophores: DAPI stain for nuclei, fibrillarin antibody (anti-fibrillarin) and ethidium bromide (EtBr) for staining nucleoli. Since the nucleolus contains very few pixels, we study only the DAPI images. Each volumetric image of both cell lines is in 3D TIFF format and has dimensions $1024 \times 1024 \times Z$ voxels, where *Z* ranges from 30 to 40 slices in the fibroblast cell collection and 65–80 slices in the PC3 cell collection. The voxel size of all volumetric images from both sets is $0.1318 \times 0.1318 \times 1$ $\upmu {\text{ m }}^3$. The dataset also includes meta-data extracted from the original data. It comprises class labels that include base filename with channel codes, information about fluorophores, stain protocol, dimensions of subvolume and references of work where data is previously published.

### Data preprocessing and segmentation

As a first step, segmentation of individual cell nuclei from potentially noisy microscopic images is performed before classification. At times, uneven staining may cause inaccurate segmentation, therefore as a quality control protocol, nuclei at the border of the image or without nucleoli are excluded. Slices from the volumetric image are extracted using ImageJ^35^ and fed into CellProfiler^36^. The CellProfiler modules (see Supplementary Fig. S3a) identify the nuclei and nucleoli and relate them, followed by filtering out of unwanted nuclei. Considering uneven illumination in the images, adaptive thresholding with Otsu’s method^37^ is employed. The approach classifies image pixels into three classes, namely background, foreground and middle intensity, where the middle intensity has the option to be merged into the foreground or background in the final segmentation result. The CellProfiler output window shows corresponding results that are promising for closely positioned objects (see Supplementary Fig. S3b). In addition, instances of connected nuclei are resolved by applying the watershed algorithm^38^ wherever required (see Supplementary Fig. S3c).

Following segmentation, cell objects are cropped from the image slices and stacked to form a 3D cell object for classification. The average dimension of cropped cell objects across all samples is $64 \times 64$ pixels. Due to parameter setting for the Otsu algorithm, objects with very low intensity could not be identified in a few top-most and bottom-most slices, resulting in final object thickness up to 15 slices for fibroblast cells and 60 for PC3 cells.

A comparison of the achieved segmentation results with the SOTA works^10,11^ on the same dataset is provided in Supplementary Table S1. In the current results, 22 more *SS* cell objects, 25 fewer *PROLIF* cell objects, 103 and 141 more *EMT* and *EPI* cell objects are detected, respectively. According to Kalinin et al.^10^, their segmentation results do not represent ground truth, as they are not hand labelled by an expert. Their study discarded connected cell objects; however, this study employed a semi-automated approach where results from CellProfiler are visually inspected, and the watershed algorithm is applied to segment connected cell objects, leading to a higher number of SS, *EMT* and *EPI* cell objects. CellProfiler, being a highly sensitive tool, has discarded comparatively more *PROLIF* cell objects, based on applied quality protocols.

### SRP and 3D SRP

SRP is a rotationally invariant texture feature descriptor, which describes the distribution of intensities in diverse patterns. It is defined by five functions computed by accessing image pixels in a global, circular and square pattern, and pixel differences in an angular and radial pattern. Values from each ring are sorted, concatenated and projected to a lower dimensional space while preserving the original distances between data points^14^. Dimensionality reduction is performed via RP which projects points from a high-dimensional space to a randomly created lower dimensional space using the *RP* matrix and a linear operation.

RP is designed based on the theory of compressed sensing, which states that any non-adaptive linear measurement in the form of random projections is capable of preserving intrinsic and salient information of the original compressible signal in lower dimensional space^14^. *RP* matrices can be constructed in various ways. In this work, the method employed follows Medeiros et al.^39^ where the *RP* matrix elements $(rp)^{ij}$ are obtained randomly as

5 $$\begin{aligned} rp^{ij}= & {} {\left\{ \begin{array}{ll} +1, &{}\quad \text {with probability 1/2,}\\ -1, &{}\quad \text {with probability 1/2.} \end{array}\right. } \end{aligned}$$

**Figure 5:**
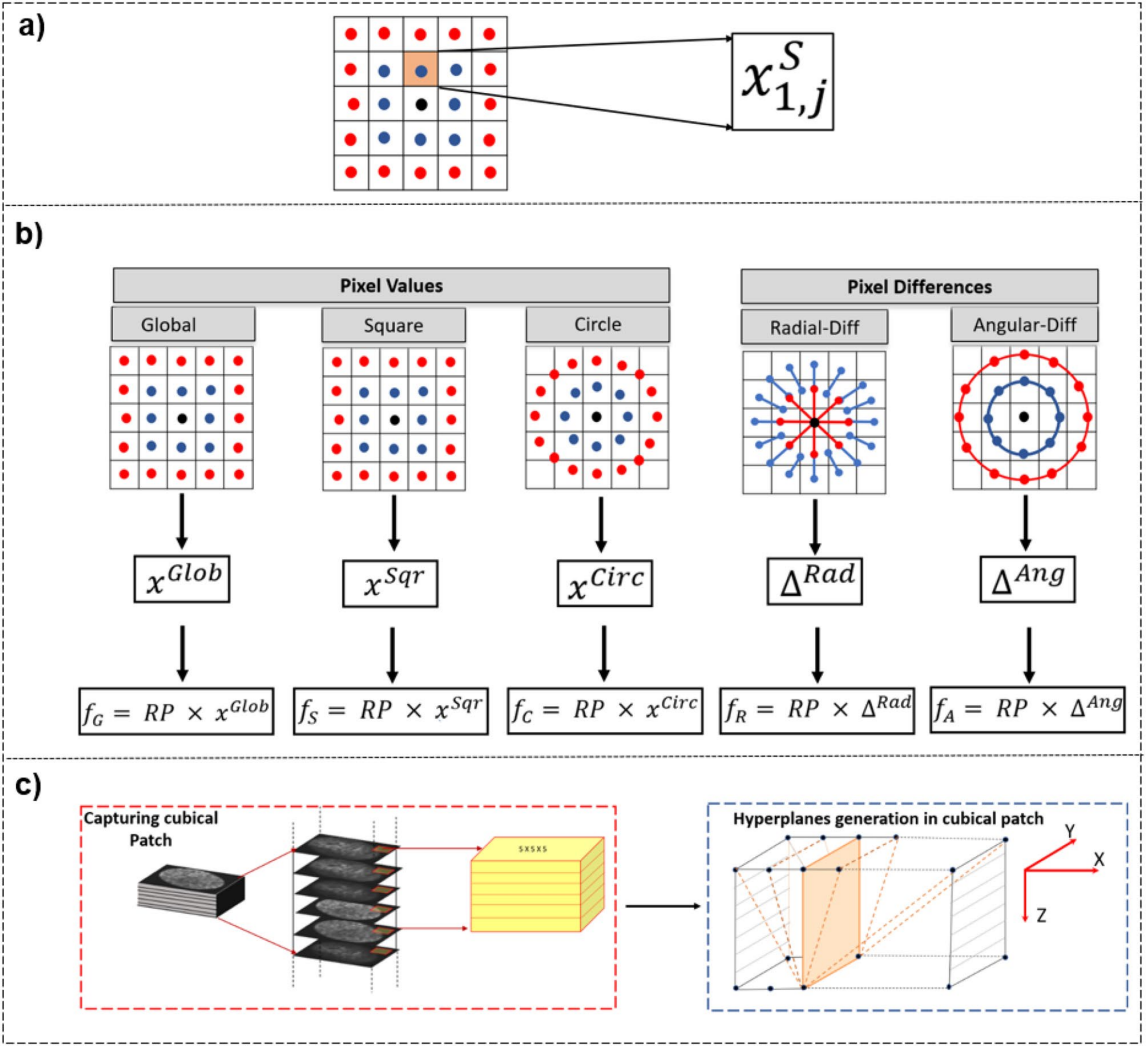
SRP. (**a**) Represents pixel value in 1*st* Square ring (*S*) at *j* *th* position. (**b**) Pixel values and differences are extracted in five different patterns from image patch size of $5 \times 5$ pixels followed by computation of local sorted descriptors (${x}^{Glob}$, ${x}^{Sqr}$, ${x}^{Circ}$, $\Delta ^{Ang}$ and $\Delta ^{Rad}$) using Eq. (7). Feature functions ($f_{S}$, $f_{G}$, $f_{C}$, $f_{A}$ and $f_{R}$) are obtained and horizontally concatenated to build SRP feature descriptor. (**c**) Proposed 3D SRP approach.

Accordingly, the high dimensional data vector *D* of dimension *b* is transformed to lower dimensional space *L* utilising the *RP* matrix with dimensions $a \times b$ and simple linear operation:

6 $$\begin{aligned} L= RP \times D \end{aligned}$$

where the resultant dimension of *L* is *a*. The value of *a* in the dimension of RP is correlated with the patch size of the image. As SRP features are extracted in concentric circles and squares (Fig. 5b), options for patch size dimensions are odd numbers as $[(2n+1) \times (2n+1)]$ where *n* = 2,3,...*N* and depend on the original dimensions of the input image and the level of the desired description. The value of *a* equals 10 for *n*=2 and for every increment in *n*, *a* increases by 10. These are the optimal RP dimensions for corresponding patch sizes and have been empirically confirmed and presented by Liu et al.^40^ under different normalisation techniques.

As illustrated in Fig. 5a, $x^S_{i,j}$ is the pixel in the *i*th square ring (*S*) in *j*th position, ${x}^{Sqr}$ is the final vector built by concatenating sorted pixels of *m* concentric squares and $p_{i}$ is the number of pixels in the *i*th square. This is represented as

7 $$\begin{aligned} {x}^{Sqr}=[{x}_{0,0}; sort([{x}^{S}_{1,0};{x}^{S}_{1,1};\ldots ;{x}^{S}_{1,p_{1}-1}]);\ldots ;sort([{x}^{S}_{m,0};{x}^{S}_{m,1};\ldots ;{x}^{S}_{m,p_{m}-1}])] \end{aligned}$$

Similarly, ${x}^{Circ}$, $\Delta ^{Ang}$ and $\Delta ^{Rad}$ are computed for pixels in circular pattern, angular pixel difference, and radial pixel difference, respectively. ${x}^{Glob}$ is obtained by sorting all the pixels of the patch. Accordingly, the feature vector of a square pattern $f_{S}$ is computed through a linear operation:

8 $$\begin{aligned} f_{S}= RP \times {{x}}^{Sqr} \end{aligned}$$

Similarly, $f_{G}$, $f_{C}$, $f_{A}$ and $f_{R}$ are computed for global, circular, angular and radial patterns, respectively. The angular and radial differences between pixels preserve the inter-distance of neighbouring pixels, making SRP feature description invariant to rotations.

Unlike other texture descriptors utilised in this study, there is currently no 3D version of SRP. To extract 3D SRP features, the existing 2D SRP is thus extended and the third plane information is proposed to be accessed using cubic patches and building multiple hyperplanes within the patch. For 3D SRP, as shown in Fig. 5c, a cubic patch is extracted from a 3D volumetric image with dimensions $5 \times 5 \times z$, where *z* in this implementation is 5, the same value as the dimensions in *X* and *Y* planes. For images with slices fewer than $[2(2n+1)]$, *z* can be the same as the number of slices. As demonstrated in Fig. 5c, multiple hyperplanes are generated between each column of the bottom-most slice and columns of the top-most slice in a cubic patch. Pixel coordinates between the top and bottom slices are identified using 3D Bresenham’s line algorithm. Five SRP functions for each hyperplane ($f_{{G}_{h}}$, $f_{{C}_{h}}$, $f_{{S}_{h}}$, $f_{{A}_{h}}$, $f_{{R}_{h}}$) are computed and concatenated across all hyperplanes built along the *YZ* plane of the cubic patch.

The resultant vectors $[f_{G}]_{CP_{yz}}, [f_{C}]_{CP_{yz}}, [f_{S}]_{CP_{yz}}, [f_{A}]_{CP_{yz}}, [f_{R}]_{CP_{yz}}$ (*CP* stands for cubic patch) are each normalised into 16 bin histograms and combined to represent the SRP descriptor of the *YZ* plane of a cubic patch with dimension 80 (16 bins $\times$ 5 functions):

9 $$\begin{aligned} SRP_{CP_{yz}}= [norm([f_{G}]_{CP_{yz}}),norm([f_{C}]_{CP_{yz}}),norm([f_{S}]_{CP_{yz}}),norm([f_{A}]_{CP_{yz}}),norm([f_{R}]_{CP_{yz}})] \end{aligned}$$

This process is repeated for the *XZ* plane as well. Features from the *XY* plane are extracted from the slices following the same computational sequence. Subsequently, descriptors from the three planes are concatenated to obtain the final 3D SRP feature descriptor for a cubic patch with dimension 240 (80 bins $\times$ 3 planes):

10 $$\begin{aligned} SRP_{CP} = [SRP_{CP_{yz}},SRP_{CP_{xz}},SRP_{CP_{xy}}] \end{aligned}$$

The patch-based feature description results in a large set of local feature descriptors of cubic patches which are often redundant and could deteriorate the classification performance. Since SRP features are generated from the RP matrix, they are random and already globalised. However, their coupling with non-local statistical feature descriptors such as BOVW can make them robust and improve their classification performance. Hence following BOVW, feature vectors of cubic patches from all volumetric images are clustered to generate a visual vocabulary of the most informative and dominant features by utilising *k*-means and sum pooling (details in the “Feature representation” section). Subsequently, each 3D image is represented by the visual vocabulary word that is identified based on the closest distance between the image feature and visual words in the codebook. In this way, the volumetric image represented by a set of cubic patches is embedded into a compact vector space.

### Other texture descriptors

Implementation details of other handcrafted feature descriptors is provided in “Supplementary Information S1”.

### Deep learning

In this study, deep learning features are also evaluated by applying the transfer learning technique. Transfer learning reduces the complexity of obtaining deep learning features and has shown success in various biomedical studies either by fine-tuning the network on available data or as a feature extractor. With VGG-16 (a CNN model trained on ImageNet), the input image goes through a series of convolutional layers before it finally produces a dense set of local feature descriptors of 512 dimensions at the last fully convolutional layer. The typical usage of a pre-trained CNN model for classification is to derive 4096-dimensional CNN features from the penultimate fully-connected layer, namely C-FC. In the current study, the local features of the last convolutional layer from all slices of the 3D image are generated with the pretrained VGG-16 model. Obtained features are further encoded into a Fisher Vector (FV)^41,42^ which produces the resultant CNN based FV descriptor (FV-CNN) with dimension 65536 (details in the “Feature representation” section).

The ImageNet pretrained VGG-16 model has established itself as a reliable choice in biomedical studies^43^. Using it directly for feature extraction does not demand any changes to the architecture by the user and provides robust deep learning features^44,45^. This study utilises FV-CNN features only to estimate the performance of the proposed approach with respect to the deep learning features. While the usual practice to obtain CNN features in biomedical studies is by training a new CNN model, the available number of images poses a limitation on training an optimal CNN. Since this study is not focussed on CNN design, experiments with a pre-trained CNN model are conducted instead.

### Feature representation

To overcome high intraclass and low interclass variations, robust texture description is required, alongside compact and efficient representation to make it useful in real life applications. Therefore feature representation techniques such as BOVW^15^ and FV^41,42^ encoding are employed which group local feature descriptors into elements of a codebook that encodes many redundant local features into high-level features.

The current implementation of BOVW utilises *k*-means clustering for codebook generation, an unsupervised learning algorithm to generate the visual vocabulary as clusters, and sum pooling to quantise the image in the form of a histogram vector. Before codebook generation, an appropriate value of *k* is obtained following the elbow method. In this method, *k*-means clustering is performed and the Sum of Squared Error (SSE) is obtained for different values of *k* (32, 64, 96, 128, 160). A graph of SSE for each value of k is plotted, which usually takes the shape of an arm, and the value of k corresponding to the elbow of the arm is chosen as an optimal value which represents the least value of *k* after which SSE scarcely varies. BOVW encoding is applied to the pseudo and non-pseudo versions of SRP, LBP, SIFT and RSurf and the resultant feature dimension is 64.

The FV descriptor encoding employs Gaussian Mixture Models (GMM)^45^, a probability density function for codebook generation. Unlike BOVW where the image is represented by the number of occurrences of the visual word, FV encodes the gradient of the log likelihood of features with respect to the GMM parameters (mean vector, standard deviation vector and mixing weights). The current implementation generates G = 64 Gaussian components from patch-level features. The final FV descriptor of each image is the concatenation of the derivatives with respect to GMM parameters, resulting in the dimension of the FV encoding of each image as 2GD, where D is the dimension of the local feature vector. This FV encoding is applied to the deep learning (CNN) features, and the resultant dimension of FV-CNN is 65536 ($2 \times 64 \times 512$). Considering the high dimension of the FV encoding, we used BOVW for handcrafted features. FV for deep learning features is employed following the work of Song et al.^45^.

### Classification

SVM classification model is trained on the implemented texture feature descriptors, utilising the Radial Basis Function (RBF) as the kernel function for automatic cell nucleus classification. *SS* and *EMT* are labelled as the positive group; *PROLIF* and *EPI* represent the negative group.

### Cross-validation

In order to evaluate the performance of cellular textural feature descriptors for volumetric image-based classification, 10-fold cross-validation is used. Each dataset is split at the 3D image level into ten subsets, ensuring that all cells from the same 3D image are in the same subset. There are 45–50 fibroblast cells and 25–45 PC3 cells per subset and performance evaluation of the proposed model is carried out in ten iterations. Following a standard cross-validation setup^46^, in each iteration, one subset is held out for model testing (an independent test set), and the remaining nine subsets are used to train the model (outer training set). To train the SVM classifier using the outer training set, 100 random samples within the outer training set are used to tune the RBF kernel parameter (gamma) following a heuristic search method^47^. The outer training set is then used to train the SVM classifier with the optimised gamma parameter and default regularisation parameter *c*. The trained model is tested on the holdout test set. This process is repeated ten times by rotating the sampling of the holdout set, so that the entire dataset are tested with the 10-fold cross-validation.

### Measurement of changes in chromatin pattern

The maximum value of the SRP feature descriptor functions computed from each $5 \times 5 \times 5$ cubic patch is employed to estimate the threshold to identify HC. It is followed by computing the ratio of HC to EC corresponding to the respective pixel values and pixel differences obtained from SRP functions. The patch size can be determined according to the respective classification performance.

- *HC intensity measure* HC/EC ratio based on pixel values estimates the fraction of the HC intensity in the nucleus. Following Eq. (8), for an image with *n* cubic patches, the global SRP descriptor for the *i* *th* patch is computed as [$f_{G}]_{CP_{i}}$, and the corresponding maximum value is obtained as ${m_i}$. The threshold to identify intensity corresponding to HC is taken as the least ${m_i}$ of all the respective values of the cubic patches of the volumetric image. There are cells oriented at a certain angle which makes ${m_i}$ for corner patches equal to zero, hence the threshold is set as the minimum non-zero value. The global feature descriptor vectors obtained from *n* cubic patches of the nuclear image [$f_{G}]_{Nucleus}$ are traversed, and the values above the threshold are identified corresponding to HC ([$f_{G}]_{HC}$). Subsequently, the fraction of the HC intensity in the nucleus is estimated using the following equation:
  11 $$\begin{aligned} \frac{HC}{EC} =\frac{sum([f_{G}]_{HC})}{sum([f_{G}]_{Nucleus}) - sum([f_{G}]_{HC})} \end{aligned}$$
- *HC aggregation measure* HC/EC ratio based on pixel differences estimates the fraction of the HC aggregates in the nucleus. The increase or decrease in image gradient leads to the corresponding rise or drop in the slope of the curve of HC aggregate, which in turn indicates condensation or decondensation of chromatin, respectively. As described in the SRP section, the radial and angular descriptor function for the *i* *th* patch of an image with n cubic patches is computed as [$f_{R}]_{CP_{i}}$ and [$f_{A}]_{CP_{i}}$, respectively. Following this, the maximum value of the concatenated vector ([$f_{R,A}]_{CP_{i}}$), is obtained as ${m_i}$. Similar to the intensity measure, the threshold to identify image gradient corresponding to HC is taken as the least ${m_i}$ of all the respective values of the cubic patches of the volumetric image. The radial and angular feature descriptor vectors obtained from *n* cubic patches of the nuclear image ([$f_{R,A}]_{Nucleus}$) are traversed, and the values above the threshold are identified corresponding to HC ([$f_{R,A}]_{HC}$). Subsequently, the fraction of the HC aggregates in the nucleus is estimated using the following equation:
  12 $$\begin{aligned} \frac{HC}{EC} =\frac{sum([f_{R,A}]_{HC})}{sum([f_{R,A}]_{Nucleus}) - sum([f_{R,A}]_{HC})} \end{aligned}$$

We note that the proposed method to compute HC/EC can also be applied to 2D images. Standard SRP functions can be computed from the 2D images following Eqs. (7) and (8). Similar to the 3D approach, the threshold to identify HC in 2D images is also the maximum value of the SRP function vector, and thus the ratios can be computed using Eqs. (11) and (12).

## Conclusions

This work proposes a new approach to extract 3D texture, where multiple hyperplanes are built within the cubic patch to extract 3D SRP features. The proposed method is utilised to classify nuclear morphology and measure variations in heterochromatin intensity and aggregation (coarsening or opening of heterochromatin) in DAPI images. The method evaluates changes in intensity values and differences between the two phenotypes of human fibroblast and human prostate cancer cell lines obtained from SOCR. Performance improvement is achieved over state-of-the-art classification, demonstrating the significance of texture features compared to shape features in the nuclear morphological study. The results also suggest that heterochromatin undergoes considerable change in intensity and aggregation on the transition from normal to another phenotypic state in fibroblast and PC3 cells. Furthermore, two different cellular states in EMT class are identified and characterised, which are considered to hold critical biological significance when studying cancer progression and drug resistance. As further application of our method, it will be interesting to see the results of the proposed HC/EC computation on the 2D fluorescence microscopy images of cells such as white blood cells obtained from a liquid biopsy.

## Supplementary information

Supplementary Information 1.

## Data Availability

Available online on the project webpage SOCR 3D Cell Morphometry Project (2018), http://socr.umich.edu/projects/3d-cell-morphometry.
