# Supplementary material for: Estimation of three-dimensional chromatin morphology for nuclear classification and characterisation
Source: Sci Rep. 2021 Feb 9;11:3364. doi: 10.1038/s41598-021-82985-9 (PMC7873284; doi:10.1038/s41598-021-82985-9)
Supplement: Supplementary file 1 — Supplementary Information 1. [file 41598_2021_82985_MOESM1_ESM.pdf]

Estimation of Three-Dimensional Chromatin Morphology for Nuclear Classification and Characterisation

Priyanka Rana[1], Arcot Sowmya[1], Erik Meijering[1,2], and Yang Song[1,*]

[1]School of Computer Science and Engineering, University of New South Wales, Sydney, New South Wales, Australia
[2]Graduate School of Biomedical Engineering, University of New South Wales, Sydney, New South Wales, Australia
[*]yang.song1@unsw.edu.au

Supplementary Information

Implementation details of 3D SRP and classification pipeline. The dataset were downloaded from http://socr.umich.edu/projects/3d-cell-morphometry. Images are in 3D TIFF format and of size $1024 \times 1024 \times Z$ voxels, where Z ranges from 30 to 40 slices in the fibroblast cell collection and 65 to 80 slices in the PC3 cell collection. 2D image slices of size 1024×1024 pixels are resized to 256×256 pixels for processing. As detailed in the Data Preprocessing and Segmentation section, cells with nucleoli are identified, segmented and cropped using CellProfiler. Cellular texture features are then computed using 3D SRP from image patches, each of $5 \times 5 \times 5$ voxels. The parameter a (number of rows of random projection matrix) correlates with the patch size and is set to 10 based on the empirical analysis. The derived feature vectors are each normalised into 16 bin histograms and combined from the three planes. The 3D SRP feature vector is of dimension 240 (16 bins x 5 feature vectors x 3 planes). Bag of Visual Words is then applied to further encode the feature vector with 64 clusters.

An SVM classifier with Radial Basis Function kernel is trained on the encoded features for nucleus classification. A 10-fold cross-validation is employed to evaluate the classification performance. During each iteration of the 10-fold cross-validation, the gamma parameter for SVM is optimised on the training set ($9/10$ of the data) using a heuristic search technique; and testing is conducted on the holdout test set ($1/10$ of the data). In addition, 3D SRP features are also used to measure heterochromatin intensity and aggregation in each cell. Cubic patch size of $5 \times 5 \times 5$ voxels is used to estimate the threshold to identify heterochromatin. Changes in chromatin pattern on transition to another phenotypic state are evaluated using the Wilcoxon rank-sum test.

Implementation details of other texture Descriptors. In order to achieve a reasonable comparison, the same patch size (5x5 pixels) as 3D SRP is used for 3D LBP. RSurf features are extracted from the whole image.

LBP features for this study are computed following LBP-TOP[1], a 3D version of LBP which suggests computation of standard LBP features from three orthogonal planes separately and concatenate them to represent 3D LBP feature descriptors. Following Zhu et al.[2], neighbourhood and radius of 16 and 2 are used respectively for 5x5 patch size.

Computation of the 3D SIFT[3] feature vector utilises a spherical image window of radius 2σ (the constant multiple of different scales of the scale space pyramid) and its centre as the keypoint. To represent image data, the gradient histogram is generated for each cubical sub-region (dimensions 4x4x4 voxels) of the spherical image window. The gradient histogram for each subregion is computed with 12 directions per histogram; therefore, the dimension of the final feature vector is (4x4x4) x 12 = 768. The utilised dimensions of the cubical sub-region and histogram length in this study have been defined as the optimal parameters in the original work, which detects the maximum number of keypoints.

RSurf[4] features, one of the latest additions to the list of handcrafted features, captures intensity variations by traversing the image in different directions. The traversed pixel vector is represented by four functions (length of the vector, difference between the highest and lowest intensities, sum of all intensity values, and the number of times the intensity varied from high to low and vice versa). For pseudo 3D RSurf, the implementation in this work considered four angles (0° , 45°, 90°, 135°) to traverse the intensities in each slice. For non-pseudo 3D RSurf[5], spherical coordinates where azimuthal angle varies from 0° to 360° at intervals of 45° and polar angle from -90° to 90° at intervals of 30° have been used. Diverse values of angles enable

rotational invariance.

Supplementary Table S1: Number of 3D volumetric images in each class of Fibroblast and PC3 cell lines.
Human fibroblast cell line has two classes: *PROLIF* and *SS*. *SS* cells are obtained by subjecting fibroblasts cells to G0/G1 Serum Starvation Protocol which is known to produce changes in nuclear shape or size in human fibroblast cells[6]. Human prostate cancer cells (PC3) have cells in two states/classes: *EPI* and *EMT*.

FIBROBLAST CELL LINE		PC3 CELL LINE	
**PROLIF**	**SS**	**EPI**	**EMT**
64	112	50	51
176		101	

Supplementary Table S2: Comparison of segmentation results

Phenotype	SOTA[7,8]	Proposed Method
PROLIF	470	445
SS	498	520
EMT	148	251
EPI	310	451

Supplementary Figure S1: Representative cell nuclei from 2D image slices.
The figure displays the cropped images of cell nuclei from the slices of volumetric images in the considered SOCR dataset[7,8]. This dataset consists of two cell lines (Fibroblasts and PC3) each comprising two distinct phenotypic states: PROLIF and SS, EPI and EMT. The representative cell nuclei exhibit a visible difference in the overall intensity distribution between the phenotypes within the same cell type. Heterochromatin can be identified in DAPI images corresponding to high luminance contrast regions. The current study encodes the intensity distribution using the proposed 3D SRP to capture variations in the image intensity that form nuclear texture features for classification of cellular phenotypic states.

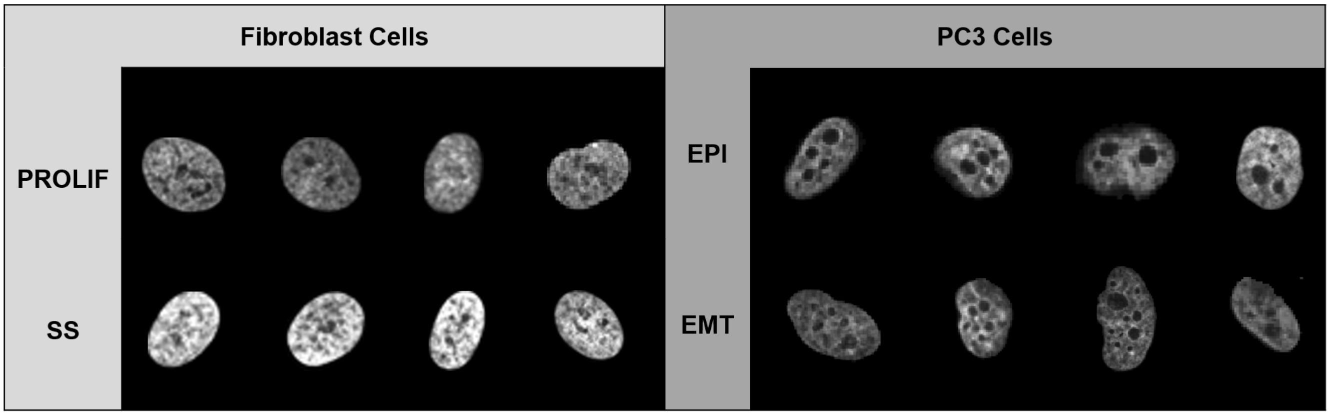

Supplementary Figure S2: 3D volumetric images of representative cell nuclei.
3D view shows evident variations in chromatin aggregation (coarsening or opening of heterochromatin) between cellular phenotypic states of each cell line that are apprehended by computing the image gradient using 3D SRP.

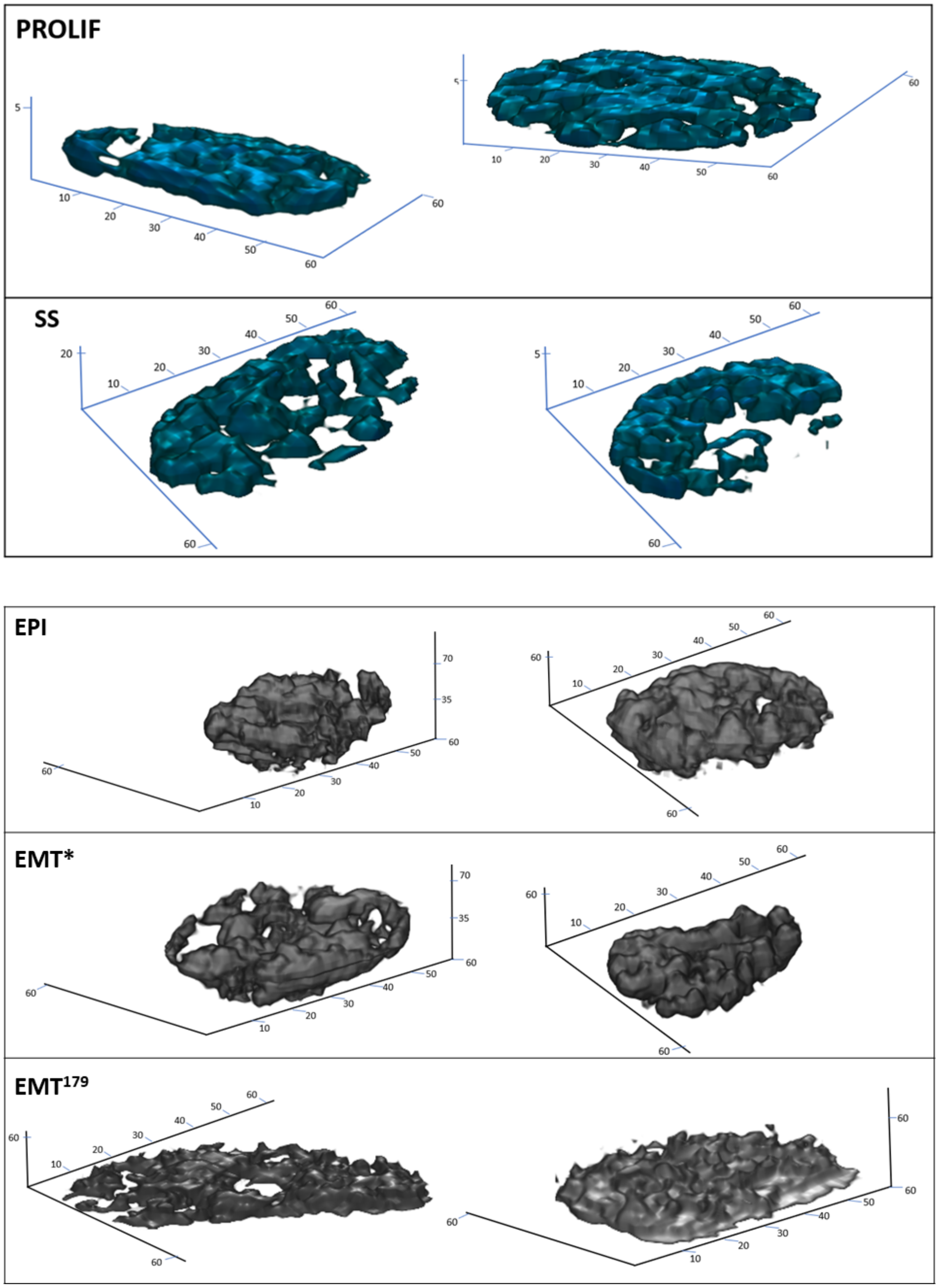

Supplementary Figure S3: Segmentation Approach.

(a) Framework of CellProfiler modules. **(b)** CellProfiler output window for segmentation. **(c)** Example Image; corresponding mask; segmentation by watershed algorithm.

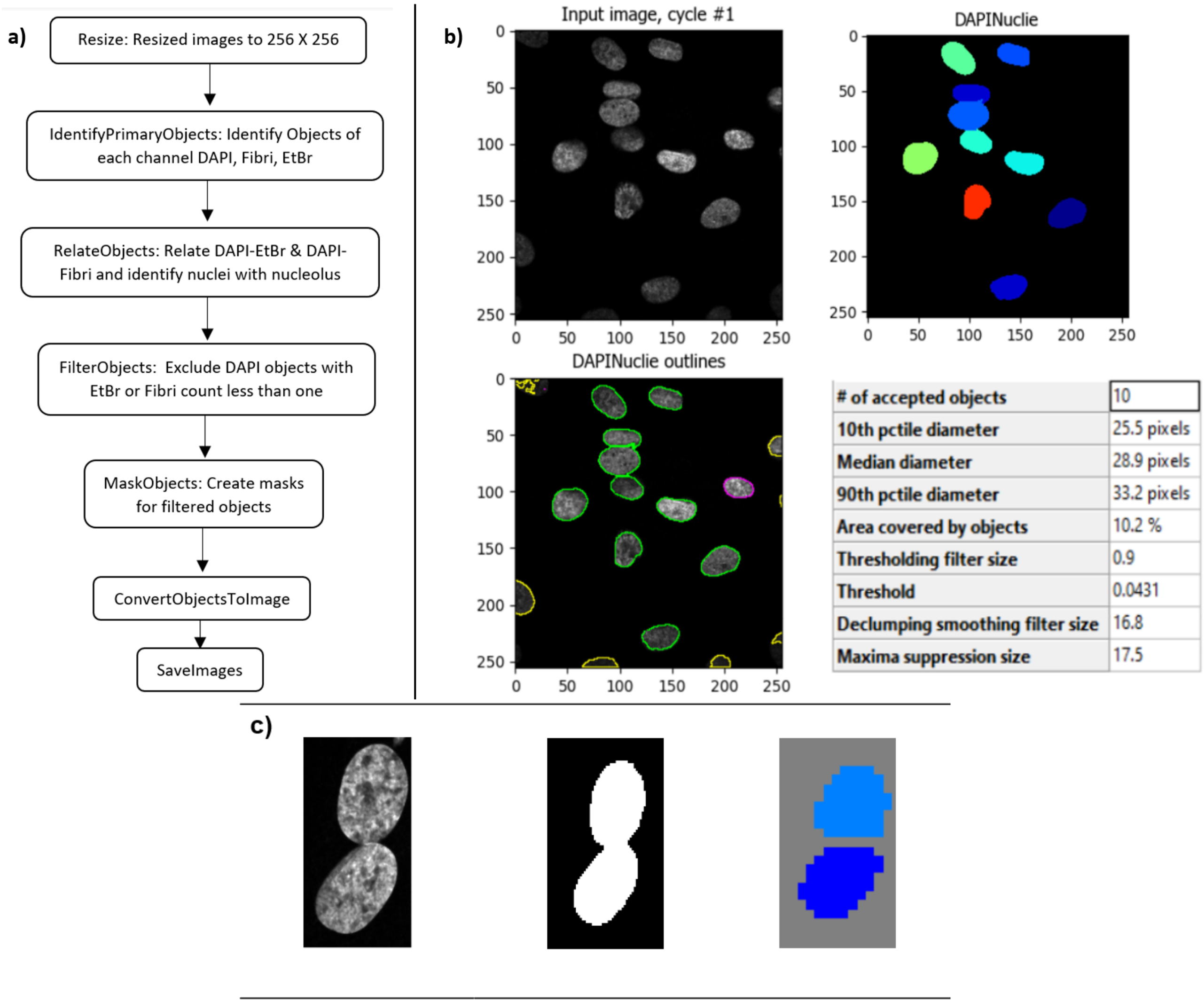

# of accepted objects	10
10th pctile diameter	25.5 pixels
Median diameter	28.9 pixels
90th pctile diameter	33.2 pixels
Area covered by objects	10.2 %
Thresholding filter size	0.9
Threshold	0.0431
Declumping smoothing filter size	16.8
Maxima suppression size	17.5

Supplementary Figure S4: ROC curves. Fibroblast Dataset.

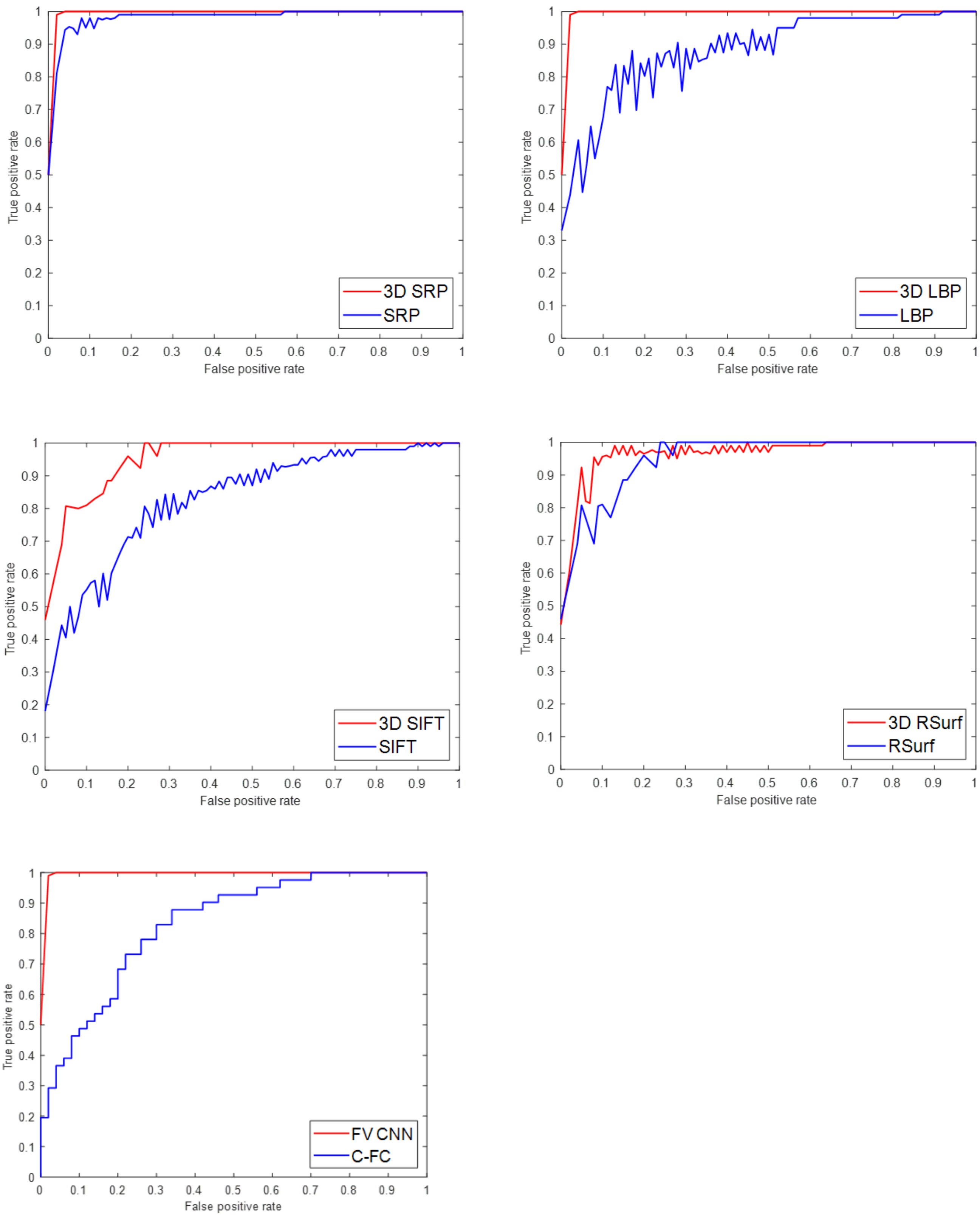

Supplementary Figure S5: ROC curves. PC3 Dataset.

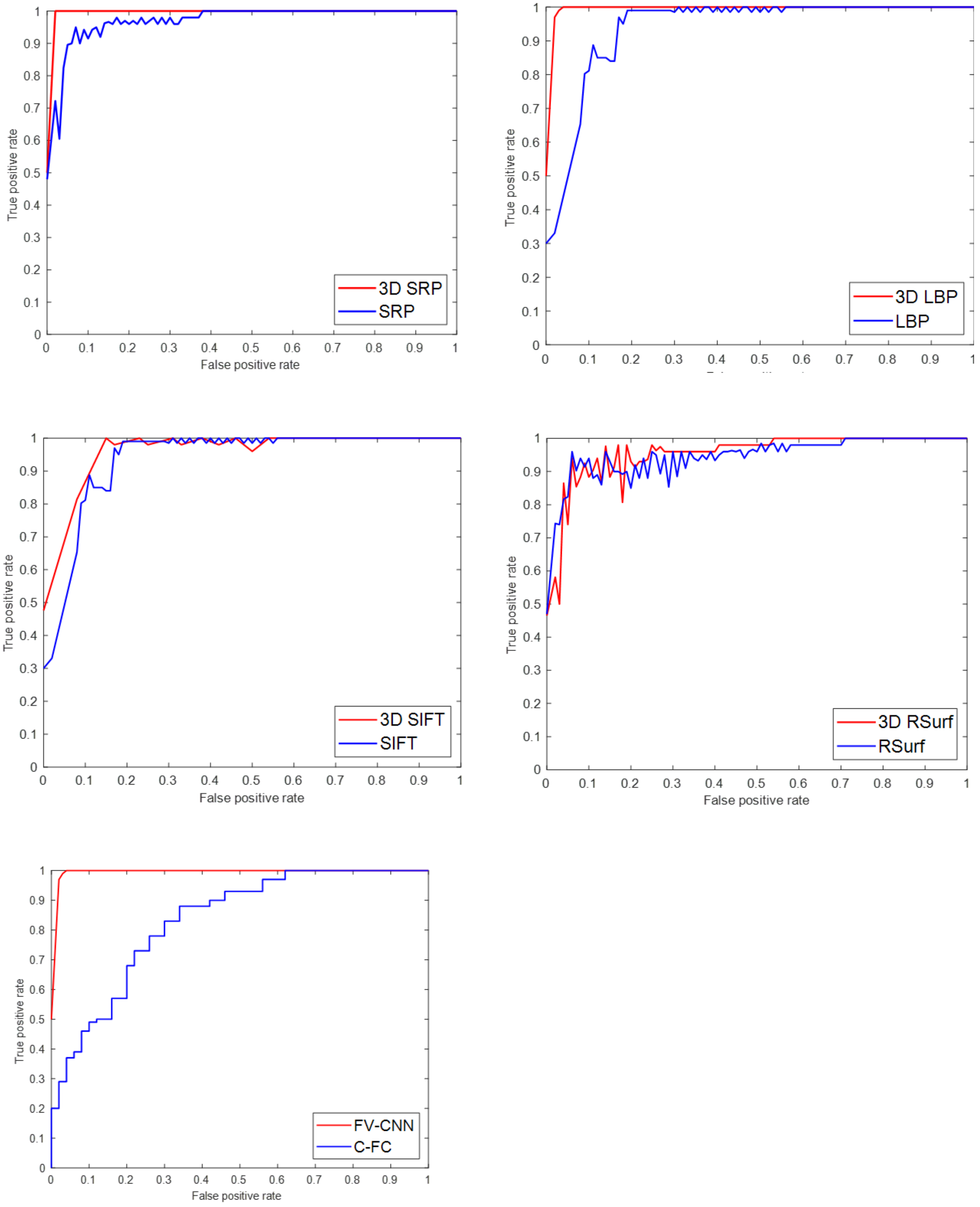

Supplementary Figure S6: Box Plots for HC/EC ratio.

Demonstration of the difference in medians of $HC/EC_{PixelValues}$ and $HC/EC_{PixelDifferences}$ between two classes. As defined in the main text, EMT^{179} refers to images from subset 179 and EMT^{*} refers to the image set without EMT^{179}. Box plots indicate both ratios are higher for SS cells than for PROLIF (normal state) cells, while they are lower for EMT cells than for EPI (normal state) cells. Since none of the notches of boxplots inside the figure overlap, indicating that HC/EC ratios of two classes are from different populations. It is also noticed that the distributions of higher HC/EC ratios and lower HC/EC ratios are similar for fibroblasts and PC3 cells.

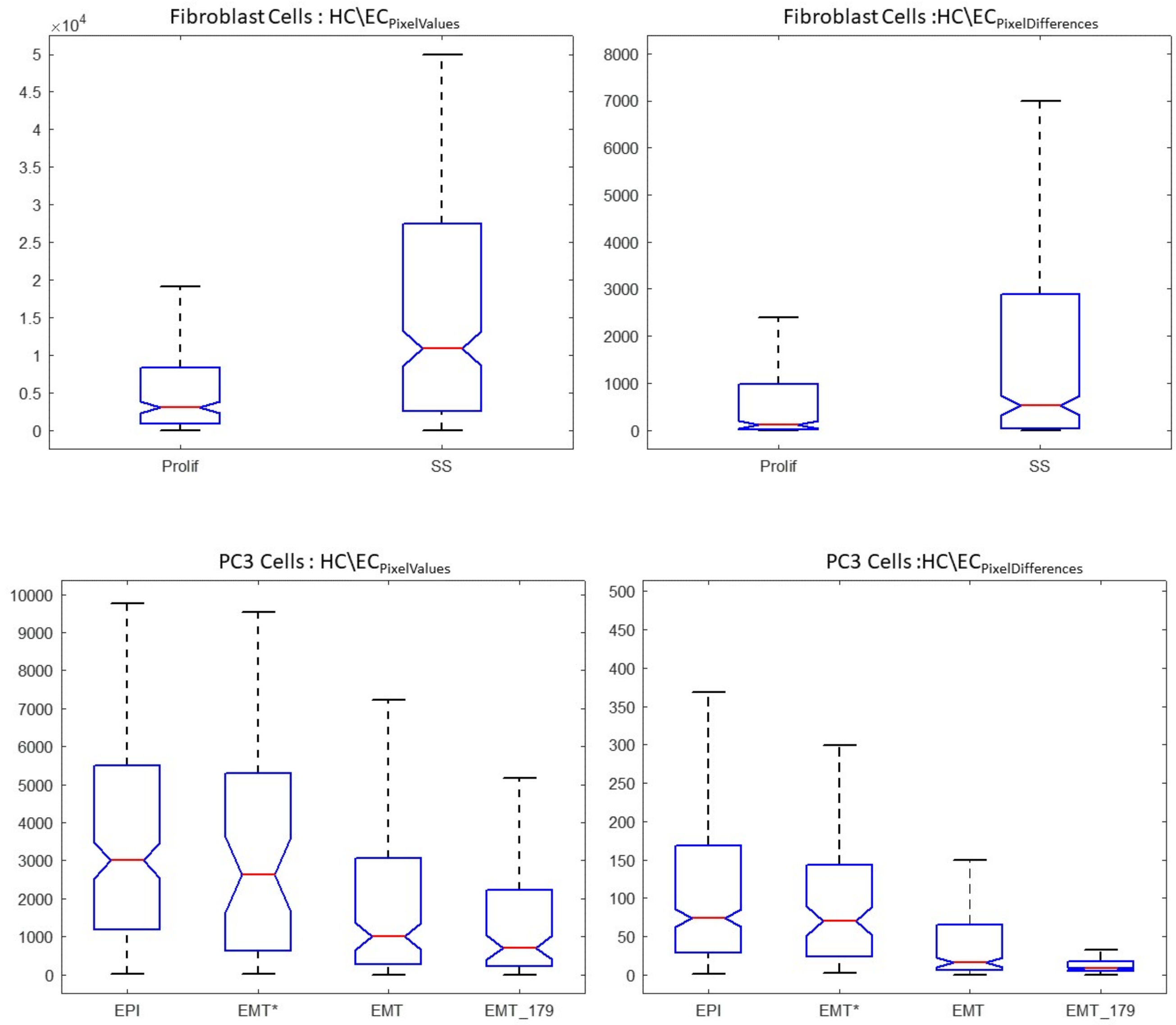

References

1. Zhao, G. & Pietikainen, M. Dynamic texture recognition using local binary patterns with an application to facial expressions. *IEEE transactions on pattern analysis machine intelligence* **29**, 915–928 (2007).

2. Zhu, C., Bichot, C.-E. & Chen, L. Multi-scale color local binary patterns for visual object classes recognition. In *2010 20th International Conference on Pattern Recognition*, 3065–3068 (IEEE, 2010).

3. Rister, B., Horowitz, M. A. & Rubin, D. L. Volumetric image registration from invariant keypoints. *IEEE Transactions on Image Process.* **26**, 4900–4910 (2017).

4. Majtner, T., Stoklasa, R. & Svoboda, D. Rsurf: the efficient texture-based descriptor for fluorescence microscopy images of hep-2 cells. In *2014 22nd International Conference on Pattern Recognition*, 1194–1199 (IEEE, 2014).

5. Stoklasa, R. & Majtner, T. Texture analysis of 3d fluorescence microscopy images using rsurf 3d features. In *2016 IEEE 13th International Symposium on Biomedical Imaging (ISBI)*, 1212–1216 (IEEE, 2016).

6. Seaman, L., Meixner, W., Snyder, J. & Rajapakse, I. Periodicity of nuclear morphology in human fibroblasts. *Nucleus* **6**, 408–416 (2015).

7. Kalinin, A. A. *et al.* 3d cell nuclear morphology: microscopy imaging dataset and voxel-based morphometry classification results. In *Proceedings of the IEEE Conference on Computer Vision and Pattern Recognition Workshops*, 2272–2280 (2018).

8. Kalinin, A. A. *et al.* 3d shape modeling for cell nuclear morphological analysis and classification. *Sci. reports* **8**, 1–14 (2018).
